# Vaccination Papers Should Be in Every Number

**Published:** 1898-06

**Authors:** B. Fincke

**Affiliations:** Brooklyn


### VACCINATION PAPERS SHOULD BE IN EVERY NUMBER.

BROOKLYN, April 30th, 1898.

WALTER M. JAMES, M. D., Phila.

DEAR DOCTOR:—Every good homœopathician is grateful for your publication of the extracts from the report of the Royal Commission on Vaccination, and I for one wish you would devote eight or ten pages in every number of THE HOMŒOPATHIC PHYSICIAN for that object. Who reads the English blue book? If those objectors do not like it, they need not read it, but the matter is of such importance that a few dissenters in our ranks do not count.

Yours fraternally,

B. FINCKE.

